# The impact of EmrA deficiency on antibiotic-susceptibility and virulence- and stone-associated phenotypes in *Proteus mirabilis*

**DOI:** 10.3389/fmicb.2026.1895236

**Published:** 2026-09-14

**Authors:** Xiao Juncong, Li Dongsheng, Tian Xiaoyan, Lin Dongni, Huang Lijun, Qiu Haoran, Huang Yuanyin, Lin Chengzhi, Zhang Mujie, Yin Zhiqiu, Peng Liang, Deng Xiaoyan

**Affiliations:** 1 KingMed School of Laboratory Medicine, Guangzhou Medical University, Guangzhou, China; 2 Department of Reproductive Medicine, Guangdong Medical University Shunde Women and Children's Hospital, Foshan, China; 3 Department of Clinical Laboratory, Key Laboratory of Biological Targeting Diagnosis, Therapy and Rehabilitation of Guangdong Higher Education Institutes, The Fifth Affiliated Hospital, Guangzhou Medical University, Guangzhou, Guangdong, China; 4 Institute of Medical Laboratory Big Data & Economics, Guangzhou Medical University, Guangzhou, China; 5 Engineering Technology Research Center of Intelligent Diagnosis for Infectious Diseases in Guangdong Province, Guangzhou, China

**Keywords:** efflux pump, *EmrA*, infection stone, *Proteus mirabilis*, resistance, urease, urinary tract infection

## Abstract

**Background:**

*Proteus mirabilis* is a common uropathogen responsible for urinary tract infections (UTIs). The *emrA* gene encodes the EmrA protein, which is a critical component of the EmrAB-TolC multidrug efflux pump. In addition to its established role in antibiotic resistance, emerging evidence indicates that EmrA is also implicated in bacterial stress adaptation and virulence-associated processes. Nevertheless, its specific functions in the pathogenesis of *P. mirabilis* remain unclear.

**Results:**

To define the function of EmrA in pathogenicity of *P. mirabilis*, this study combined phenotypic assays, transcriptomics, and a rat model of urinary tract infection. Comparison of the WT and Δ*emrA* strains showed that *emrA* deletion was associated with decreased MICs for carbenicillin and ampicillin, whereas the susceptibilities to most other antibiotics remained unchanged. Because MICs were not determined for the complemented strain, these results identify a deletion-associated susceptibility phenotype but do not establish an *emrA*-specific resistance mechanism. The study also found that EmrA promotes its adaptation to nutrient limitation, heat, and acid stress. However, the deletion of *emrA* significantly increased bacterial tolerance to oxidative stress. The mutant strain exhibited enhanced virulence-associated phenotypes, including increased swarming motility, biofilm formation, and improved adhesion and invasion capabilities of urothelial cells. Transcriptomic analysis revealed that *emrA* deletion upregulated multiple virulence-associated genes, including those encoding fimbriae components. In the rat UTI model, infection with the mutant was associated with higher bacterial burdens in the bladder and kidneys, and more severe inflammatory tissue damage; stone-associated outcomes were also increased in the models used. These stone-associated findings were accompanied by increased urease activity in the mutant strain.

**Conclusion:**

This study identifies associations between *emrA* deletion and susceptibility changes to selected antibiotics, altered stress responses, and enhanced virulence- and stone-associated phenotypes in *P. mirabilis*. Because MICs were not measured for the complemented strain, the specific contribution of EmrA to the antibiotic-susceptibility phenotype remains unresolved.

## Graphical abstract

**Figure:**
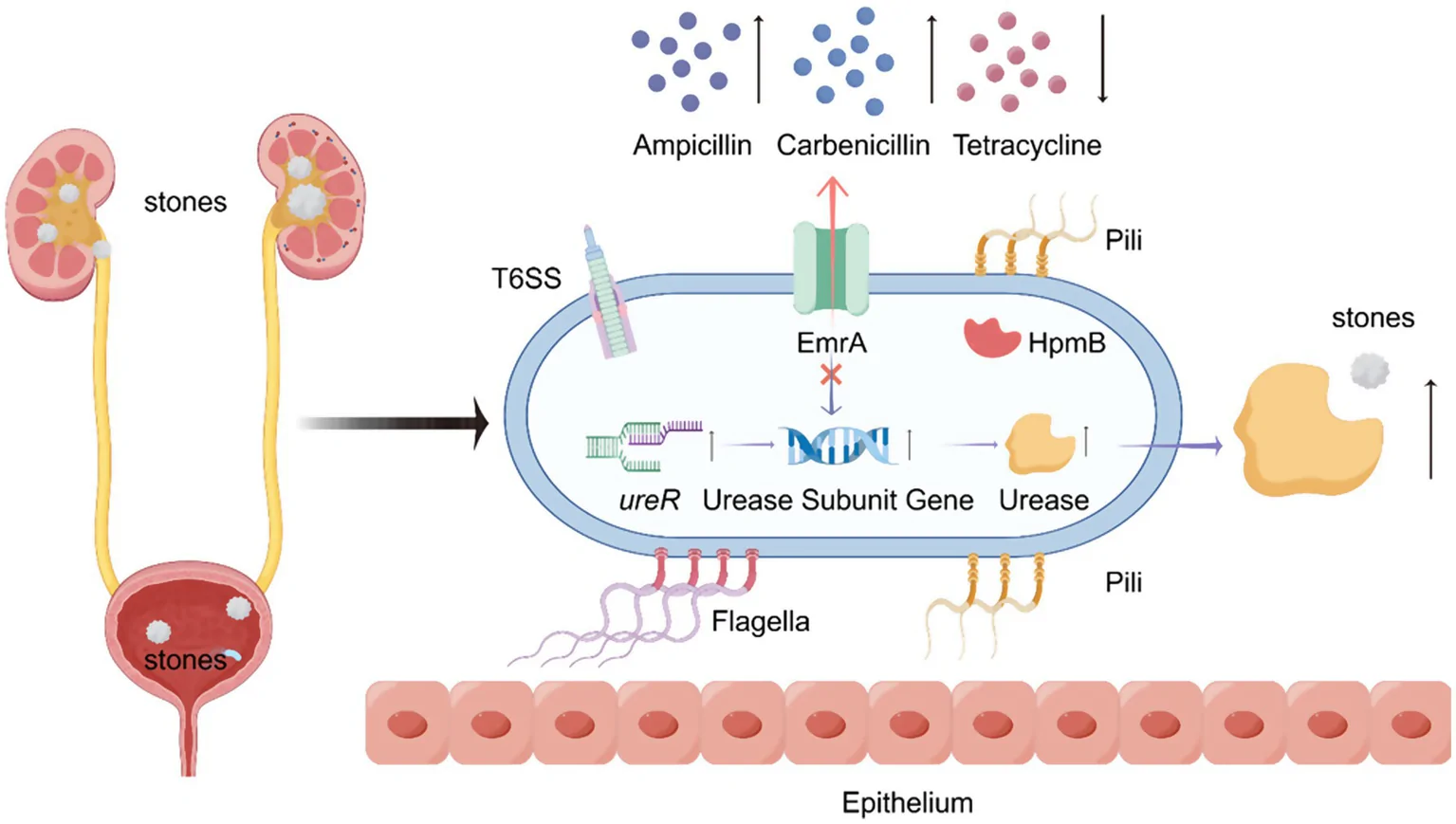

## Introduction

Urinary tract infections (UTIs) represent a major global health burden, ranking as the second most common bacterial infection after respiratory illnesses, with an estimated annual incidence affecting 150 million people (Flores-Mireles et al., 2015; Foxman, 2014). Among these, catheter-associated urinary tract infections (CAUTIs), as the most frequent hospital-acquired infections, have a mortality rate as high as 28.2%, more than twice that of non-CAUTIs (Hunt et al., 2024; Jacobsen et al., 2008). If not controlled promptly, UTIs can progress to life-threatening urosepsis. A severe complication of UTIs is the formation of infection stones, which constitute 10–15% of all urinary calculi. These stones, which can rapidly develop into staghorn calculi obstructing the renal tract, drive a cycle of urinary obstruction, hydronephrosis, and recurrent pyelonephritis, ultimately risking permanent kidney damage. Critically, bacteria within the stone matrix can evade antibiotics and host immune defense, rendering standard antimicrobial therapy ineffective and often making surgical removal necessary, despite recurrence rates of 30–50% post-operation (Schwartz and Stoller, 1999; Bichler et al., 2002; Flores-Mireles et al., 2015). The management of these complex infections is further complicated by the rising global prevalence of multidrug-resistant (MDR) pathogens (ElTaweel et al., 2024; Chakkour et al., 2024), highlighting an urgent need to develop novel therapeutic strategies.

*Proteus mirabilis i*s a major pathogen in complicated urinary tract infections (UTIs), particularly those associated with catheters (CAUTIs), where it accounts for 10–44% of isolates (Armbruster et al., 2018; Jacobsen et al., 2008). Its pathogenicity is driven by distinct virulence mechanisms, most notably its characteristic swarming motility, which facilitates rapid surface colonization and retrograde dissemination from the urethra to the upper urinary tract. Additional key virulence factors include flagella, fimbriae, hemolysin, and specialized secretion systems, which collectively mediate adhesion, invasion, toxin production, and immune evasion (Debnath et al., 2018; Pearson et al., 2008; Armbruster et al., 2018). Notably, *P. mirabilis* is among the most potent urease-producing bacteria, exhibiting significantly higher activity than other common uropathogens such as *Escherichia coli* (Mobley and Hausinger, 1989; Yang et al., 2024; Chakkour et al., 2024). This robust urease activity is the primary driver of infection stone formation, a severe complication that exacerbates disease and complicates treatment. The synergistic effect of its multifaceted virulence and stone-forming capacity solidifies its role as a major pathogen in UTIs and related urolithiasis (Norsworthy and Pearson, 2017; Flores-Mireles et al., 2015). The increasing prevalence of multidrug-resistant *P. mirabilis* strains globally further underscores the urgent need to elucidate its pathogenesis, as understanding these mechanisms is vital for developing novel therapeutic strategies (ElTaweel et al., 2024; Chakkour et al., 2024).

Bacterial efflux pumps are fundamental antimicrobial resistance mechanisms in Gram-negative bacteria, actively expelling various toxic compounds, including antibiotics. Based on structure and energy coupling, efflux pumps are classified into six major families, including the Resistance-Nodulation-Division (RND) and Major Facilitator Superfamily (MFS) groups—based on their structure and energy coupling (Li and Nikaido, 2009; Nikaido and Takatsuka, 2009; Darby et al., 2023; Li et al., 2015). Among these, tripartite RND-type pumps, which span the inner and outer membranes, are particularly clinically significant due to their broad substrate specificity. EmrA is a membrane fusion protein that functions as a critical adaptor, linking the inner membrane transporter to the outer membrane channel in efflux complexes such as EmrAB-TolC (Hinchliffe et al., 2014; Tanabe et al., 2009; Li and Nikaido, 2009). Beyond its canonical role in multidrug efflux, emerging evidence indicates that EmrA is involved in broader physiological processes, including metabolite transport, biofilm formation, heavy metal tolerance, and can influence bacterial virulence in some species (Lin et al., 2017; Ma et al., 2014). However, its specific function in the pathogenesis of *P. mirabilis*, an important uropathogen, remains unclear. Given the critical need to understand the virulence mechanisms of this pathogen, elucidating the role of EmrA in *P. mirabilis* may provide insight into the biology of *P. mirabilis* urinary tract infection and identify hypotheses for future mechanistic investigation. Our preliminary pan-genome analysis of *P. mirabilis* revealed that *emrA* gene, encoding the membrane fusion protein EmrA, belongs to the species-specific core genome, prompting the hypothesis that EmrA plays a significant role in *P. mirabilis* pathogenicity. We therefore examined the association of *emrA* deletion with antibiotic susceptibility, stress adaptation, motility, virulence-associated phenotypes, and infection stone formation. Using the clinical isolate *P. mirabilis* DP2019 as the wild-type background, we constructed an isogenic *emrA* deletion mutant (Δ*emrA*) and its genetically complemented counterpart (CΔ*emrA*). Comparative phenotypic analyses were integrated with transcriptomic profiling and RT-qPCR validation to identify pathways associated with the observed phenotypes.

## Materials and methods

### Bacterial strains and culture conditions

*P. mirabilis* DP2019 (GenBank CP110673), a clinical urinary isolate, was used as the wild-type (WT) strain. The isogenic *emrA* deletion mutant was constructed by allelic exchange using the suicide vector pCVD442Gm. Briefly, the upstream and downstream homologous fragments flanking *emrA* were amplified, fused, cloned into pCVD442Gm, introduced into the donor strain for conjugation, and transferred into *P. mirabilis* DP2019. Candidate double-crossover mutants were screened by antibiotic selection and sucrose counterselection and verified by PCR and Sanger sequencing. For complementation, the native *emrA* gene with its promoter was cloned into pRK415 and transferred into Δ*emrA* via conjugation, and CΔ*emrA* was routinely grown in Lysogeny Broth (LB) at 37 °C. Tetracycline (10 μg/mL) was used during routine culture and inoculum preparation of CΔ*emrA* for plasmid maintenance.

### Minimum inhibitory concentration

MICs were determined by the broth microdilution method in Mueller-Hinton broth (Macklin) according to CLSI guidelines (Schuetz et al., 2025). Experiments were performed in triplicate.

### Growth curve analysis

Overnight cultures were normalized to OD₆₀₀ = 0.6, diluted 1:100 into fresh LB or M9 minimal medium, and incubated at 37 °C with shaking. OD₆₀₀ was recorded every 2 h for 24 h. Oxygen-limited growth assays were performed under anaerobic conditions, and OD_600_ was recorded every 2 h for 8 h. Three independent biological replicates were performed.

### Stress survival assays

Bacterial suspensions (OD₆₀₀ ≈ 0.5 in PBS) were subjected to: (i) heat stress at 56 °C for 2 and 4 min; (ii) acid stress in LB (pH 4.0) at 37 °C for 2, 4 and 6 h; (iii) oxidative stress in 20 or 40 mM H₂O₂ at 37 °C for 15 min. Viable counts were determined by plating on LB agar. Survival rate (%) = (CFU after treatment/CFU before treatment) × 100. At least three biological replicates were performed.

### Swarming motility assay

A 10 μL aliquot of normalized bacterial suspension (OD₆₀₀ = 0.5) was spot-inoculated onto blood agar plates (1.5% agar, 5% defibrinated sheep blood). Plates were incubated at 37 °C in a humidified atmosphere. Swarming colony diameters were measured at 2-h intervals for up to 12 h. Three independent replicates were performed.

### Biofilm formation assay

Overnight cultures were diluted 1:100 in LB, and 1.5 mL aliquots were added to 24-well polystyrene plates. After static incubation at 37 °C for 24 h, planktonic cells were removed, and adherent biofilms were washed with PBS, stained with 0.1% crystal violet, and solubilized in extraction solution (ethanol: acetone, 8:2). Absorbance was measured at 570 nm. Experiments were performed in triplicate with technical duplicates.

### Cell culture and *in vitro* infection assays

Cell culture. SV-HUC-1 (urinary bladder epithelial cells) and HK-2 (renal tubular epithelial cells) were cultured in DMEM/F-12 (Gibco) supplemented with 10% heat-inactivated fetal bovine serum (Procell) at 37 °C in 5% CO₂.

Adhesion and invasion assays. Approximately 1 × 10^5^ cells/well were cultured in a 24-well plate with 1 × 10^6^ CFU/well of bacteria for 1 h. The inoculum of each strain was verified by CFU plating to ensure equivalent bacterial challenge. For adhesion, monolayers were washed and lysed with 0.5% Triton X-100. For invasion, after the 1-h co-incubation, cells were treated with gentamicin (100 μg/mL) for 1 h to kill extracellular bacteria, then lysed. Serial dilutions were plated for CFU enumeration. Adhesion and invasion rates were expressed as percentages of the initial inoculum, and mock-infected controls were included to monitor baseline host-cell viability.

Cytotoxicity assays. Approximately 1 × 10^4^ cells/well were cultured in a 96-well plate with 1 × 10^6^ CFU/well of bacteria for 2 h. Cell metabolic activity was assessed using the CCK-8 assay (NCM Biotech), and membrane damage was quantified by LDH release (Beyotime), following the manufacturers’ protocols. Cells were infected at an MOI of 100:1 for 2 h.

### Rat model of acute urinary tract infection

Six- to seven-week-old female Sprague–Dawley rats were randomly divided into four groups: PBS-injected control, wild-type DP2019, Δ*emrA* and CΔ*emrA*, with 6 rats per group. Animals were randomly assigned to treatment groups, and histological assessment was performed using consistent scoring criteria. The animals were acclimatized for 3 days and then deprived of water for 24 h prior to infection. Overnight cultures of DP2019, Δ*emrA*, and CΔ*emrA* were grown in LB broth at 37 °C with shaking at 220 rpm. Bacterial cells were harvested, washed twice with sterile PBS, and resuspended to a final concentration of 1 × 10^10^ CFU/mL. The inoculum was verified by serial dilution and plating. For inoculation, rats were anesthetized by intraperitoneal injection of 1% sodium pentobarbital (40 mg/kg). Care was taken to avoid injecting into the thoracic cavity or bladder, which could cause death or insufficient anesthesia. Once the pedal reflex disappeared, the rat was placed in a supine position on a sterile surgical drape. The bladder was gently compressed to expel residual urine, the urethral orifice was disinfected with an alcohol swab, and a sterile drape was placed to expose the urethra. A 1-mL syringe was filled with the bacterial suspension or PBS and connected to a 24-gauge intravenous catheter. The catheter was primed with the suspension, and then the syringe was reloaded to the 0.1-mL mark. The catheter tip was lubricated with sterile glycerol and gently inserted into the urethra. When resistance was felt at the pubic symphysis, the catheter was flattened and carefully advanced into the bladder. A total of 100 μL of inoculum was slowly injected. Leakage from the urethra or catheter was monitored; if any leakage occurred, the corresponding volume was supplemented. After inoculation, the catheter was withdrawn slowly to minimize leakage and allow bacterial stabilization in the bladder. The rat was kept in a supine position with its pelvis elevated for 30 s, and any further leakage was observed. The animals were returned to their cages and kept warm; water was withheld until the following morning. At 24 h post-infection, rats were euthanized by intraperitoneal injection of 200 mg/kg pentobarbital sodium. Blood, bladder and kidneys were collected. The bladder was bisected longitudinally: one half was placed in 4% paraformaldehyde for fixation, and the other half in PBS for bacterial enumeration. The kidneys were also excised from the retroperitoneal space. The left kidney was decapsulated and fixed in 4% paraformaldehyde; half of the right kidney was placed in PBS for bacterial load determination. Bacterial loads were determined by plating tissue homogenates. Cytokine levels (IL-1β, IL-6, TNF-α) in bladder homogenates and serum were measured by ELISA. Tissues were processed for H&E and immunohistochemistry.

### Vitro crystallization assays

Bacterial lysates were prepared by sonication, and protein concentrations were normalized using the BCA assay (Beyotime). For crystal formation (nucleation), 200 μL of lysate (0.5 mg/mL) was mixed with 400 μL of improved sterile artificial urine (KH₂PO₄ 1.36 g/L, NH₄Cl 0.54 g/L, urea 25 g/L, 0.1 mol/L MgCl₂ solution 100 mL, adjust to 1 L with purified water, then adjust pH to 7.0) (Sarigul et al., 2019) and incubated at 37 °C for 1 h. Crystals were counted under light microscopy (200×) in ten randomly selected fields per sample, using the same field-selection and counting criteria across all groups. For crystal aggregation, 2 mg of struvite seed crystals were suspended in artificial urine and mixed with lysate (0.3 mg/mL, 1:10 ratio). After incubation at 37 °C for 1 h, aggregates were quantified microscopically. The tight assembly of two or more individual crystals is defined as crystal aggregation. At least three biological replicates were performed.

### Urease activity assays

Urease activity was measured by three methods: (i) phenol red colorimetric assay (0.001% phenol red in artificial urine, pH 6.0, absorbance at 550 nm); (ii) pH shift assay (direct pH measurement over 6 h); (iii) indophenol blue assay (Solarbio) for ammonia quantification. Bacterial lysates were normalized to 0.3–0.5 mg/mL protein as indicated.

### RNA extraction and RT-qPCR

Total RNA was extracted using the GeneJET RNA Purification Kit (Thermo Fisher). cDNA was synthesized with the PrimeScript RT Reagent Kit (TaKaRa). RT-qPCR was performed using TB Green Premix Ex Taq II (TaKaRa) on a CFX96 Touch system (Bio-Rad). The gene gyrB was used as an internal control. Relative expression was calculated by the 2^−ΔΔCt^ method.

### Rat model of chronic infection stone formation

All animal procedures were approved by the Institutional Animal Care and Use Committee of South China Agricultural University (Approval No. GZTH2020008; approval date: 2026-03-18). Female Sprague–Dawley rats (6 ~ 7 weeks old, 180 ~ 220 g) were randomly divided into four groups (*n* = 6 per group): PBS-injected control, WT, Δ*emrA* and CΔ*emrA*. After 3 days of acclimatization, rats underwent surgical implantation of a zinc disc into the bladder. Anesthesia was induced by intraperitoneal injection of 1% sodium pentobarbital (40 mg/kg). The lower abdominal fur was shaved, and the skin was disinfected with iodophor. A suprapubic midline incision was made to expose and open the bladder, and a sterile zinc disc (4 mm diameter) was immediately inserted into the bladder through the incision. The bladder and abdominal muscle layers were closed using absorbable sutures, and the skin incision was closed with non-absorbable sutures. Ampicillin (20 mg/kg) was administered postoperatively to prevent surgical site infections. After a 7-day recovery period, rats were inoculated transurethrally with 10^8^ CFU of the respective bacterial strains, following the same procedure as described for the acute UTI model. This was designated as day 0 of infection. Booster inoculations of 10^8^ CFU and 10^9^ CFU were administered on days 6 and 12, respectively, at the same volume. On day 14 post-initial infection, rats were euthanised, and bladder stones, blood, kidney tissues and bladder tissues were collected for subsequent analyses. The excised stones were dried and weighed using an electronic balance. Bacterial loads in bladder, kidney and urine, as well as cytokine levels, were determined as described above.

### RNA isolation, library construction, and sequencing

The WT and Δ*emrA* were inoculated into LB liquid medium at a ratio of 1:100 (vol/vol) and cultured at 37 °C with shaking at 220 rpm until the mid-logarithmic phase. The bacterial cells were then harvested by centrifugation at 8,000 rpm for 20 min, washed twice with phosphate-buffered saline (PBS), and the supernatant was discarded. The resulting cell pellets were stored at −80 °C for further use. Three independent biological replicates were performed for each strain. Total RNA was extracted from bacterial samples using TRIzol® Reagent according to the manufacturer’s protocol. The concentration and purity of the extracted RNA were assessed using a NanoDrop™ 2000 spectrophotometer, RNA integrity was evaluated by agarose gel electrophoresis, and the RNA integrity number (RIN) was determined using an Agilent 2,100 Bioanalyzer. Ribosomal RNA was removed and the enriched mRNA was randomly fragmented by the addition of fragmentation buffer. First-strand cDNA was synthesized from the fragmented mRNA using random hexamer primers. For second-strand synthesis, dUTP was substituted for dTTP in the dNTP mix, resulting in the incorporation of A/U/C/G bases into the second cDNA strand. End Repair Mix was then added to convert the sticky ends into blunt ends, followed by the addition of a single adenine (A) base to the 3′ ends for adapter ligation. The second-strand cDNA containing uracil was digested with UNG enzyme, ensuring that only the first-strand cDNA was retained in the library, which was subsequently amplified by PCR. After adapter ligation, the products were purified and subjected to size selection. The size-selected products were then amplified by PCR, and the final library was obtained after purification. Library quality was assessed, and sequencing was performed on the NovaSeq platform (Illumina). The RNA-seq data have been deposited in the SRA database under accession number PRJNA1472729.

## Results

### EmrA relates to resistance to some β-lactam antibiotics and environmental stress adaptability

To examine whether *emrA* deletion was associated with altered antimicrobial susceptibility, MICs were compared between the WT and Δ*emrA* strains. Relative to WT, Minimum inhibitory concentration (MIC) experiments revealed that the Δ*emrA* mutant exhibited significantly increased sensitivity to β-lactam antibiotics, showed an 8-fold reduction in the MIC for carbenicillin and a 2-fold reduction for ampicillin. In contrast, the MIC for tetracycline increased, whereas the MIC of most other tested antibiotics remained unchanged (Table 1). These results identify an antibiotic-specific susceptibility phenotype associated with the Δ*emrA* genotype rather than a generalized change in antimicrobial susceptibility. However, because MICs were not determined for CΔ*emrA*, the present data do not demonstrate restoration of the ampicillin or carbenicillin phenotype and cannot establish that these MIC changes are specifically attributable to loss of EmrA.

**Table 1:** Determination of minimum inhibitory concentration.

Antibiotics	DP2019	Δ*emrA*
Levofloxacin	0.125 μg/mL	0.125 μg/mL
Imipenem	2 μg/mL	2 μg/mL
Ceftriaxone Sodium	<0.125 μg/mL	<0.125 μg/mL
Gentamicin	2 μg/mL	2 μg/mL
Polymyxin B	>128 μg/mL	>128 μg/mL
tigecycline	16 μg/mL	16 μg/mL
Furantoin	>128 μg/mL	>128 μg/mL
Tetracycline	32 μg/mL	64 μg/mL
Compound Sulfamethoxazole	>128 μg/mL	>128 μg/mL
Ampicillin	1 μg/mL	0.5 μg/mL
Carbenicillin	4 μg/mL	0.5 μg/mL
Nalidixic Acid	32 μg/mL	32 μg/mL
Chloramphenicol	>128 μg/mL	>128 μg/mL

Subsequently, deletion of *emrA* is associated with antibiotic-specific susceptibility changes and altered stress adaptation. The growth of the Δ*emrA* mutant was comparable to WT in nutrient-rich LB medium, but showed a significant growth defect during the log phase in nutrient-limited minimal M9 medium (Figures 1A,B). On this basis, anaerobic environment was added, and it was found that the mutant strain grew significantly slower (Figures 1C,D). Furthermore, the mutant showed markedly reduced survival under heat shock (56 °C) and acidic (pH 4.0) conditions compared to the wild-type (WT) and complemented strains (CΔ*emrA*) (Figures 1E–H). In contrast, the survival rate of the Δ*emrA* mutant under oxidative stress (20 and 40 mM H₂O₂) exhibited significantly enhanced trend (Figures 1I,J). These data indicate that EmrA contributes to adaptation to selected environmental stresses, including nutrient limitation, heat, and acid, but unexpectedly suppresses bacterial survival under oxidative conditions.

**Figure 1:**
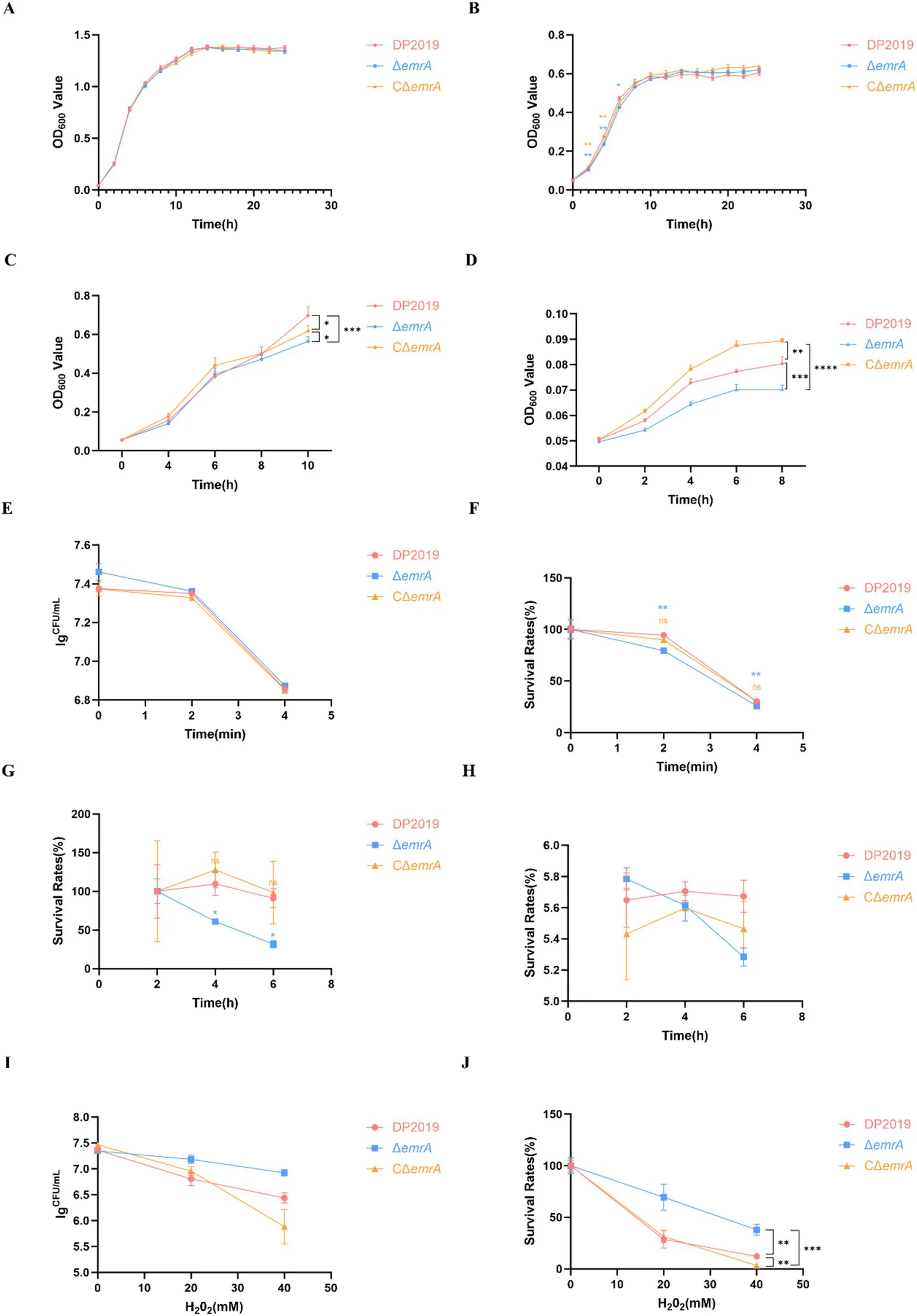
Phenotypic characterization of the Δ*emrA* mutant under stress conditions. **(A,B)** Growth curves of WT, Δ*emrA*, and CΔ*emrA* in nutrient-rich LB medium **(A)** and M9 minimal medium **(B)** (comparisons are versus the WT strain). Optical density at 600 nm (OD_600_) was measured every 2 hours for 24 h. Data are shown as mean ± SD (*n* = 3). **(C,D)** Growth curves of WT, Δ*emrA*, and CΔ*emrA* in nutrient-rich LB medium **(C)** and M9 minimal medium **(D)** (comparisons are versus the WT strain) under oxygen stress conditions. Optical density at 600 nm (OD_600_) was measured every 2 hours for 8 h. Data are shown as mean ± SD (*n* = 6). **(E,F)** Bacterial counts expressed as log_10_ CFU/mL **(E)** and corresponding survival rates **(F)** after exposure to heat stress (56 °C for 2 min and 4 min). Survival rate was calculated as (CFU after heat treatment/CFU of untreated control) × 100%. **(G,H)** Bacterial counts (log_10_ CFU/mL) **(G)** and survival rates **(H)** following exposure to oxidative stress (20 mM and 40 mM H_2_O_2_ for 15 min). **(I,J)** Bacterial counts (log_10_ CFU/mL) **(I)** and survival rates **(J)** under acid stress (pH 4 stress at 2 h and 4 h). Survival rates at 4 h and 6 h are shown relative to the 2 h time point. Statistical analysis: All experiments were performed at least three independent times with triplicate samples. Data are presented as mean ± SD. Comparisons among groups were performed using one-way ANOVA followed by Tukey’s post-hoc test (for survival rate comparisons) or two-way ANOVA with repeated measures (for growth curves). Significance levels are indicated as: * *P* < 0.05, ** *P* < 0.01, *** *P* < 0.001; n.s., not significant.

### EmrA deletion promotes a hyper-motile and hyper-biofilm-forming phenotype

Based on established connections between efflux systems and surface-associated behaviors, we investigated the role of EmrA in swarming motility and biofilm formation. Results showed that the Δ*emrA* mutant displayed a significantly larger swarming diameter than the WT and CΔ*emrA* (Figures 2A,B), demonstrating that EmrA is consistent with a negative association of this form of motility. Simultaneously, quantified by crystal violet staining revealed that the biofilm-forming ability of the Δ*emrA* mutant was significantly enhanced (Figures 2C,D). These results indicate that loss of *emrA* is associated with enhanced motility and biofilm-related phenotypes in *P. mirabilis*.

**Figure 2:**
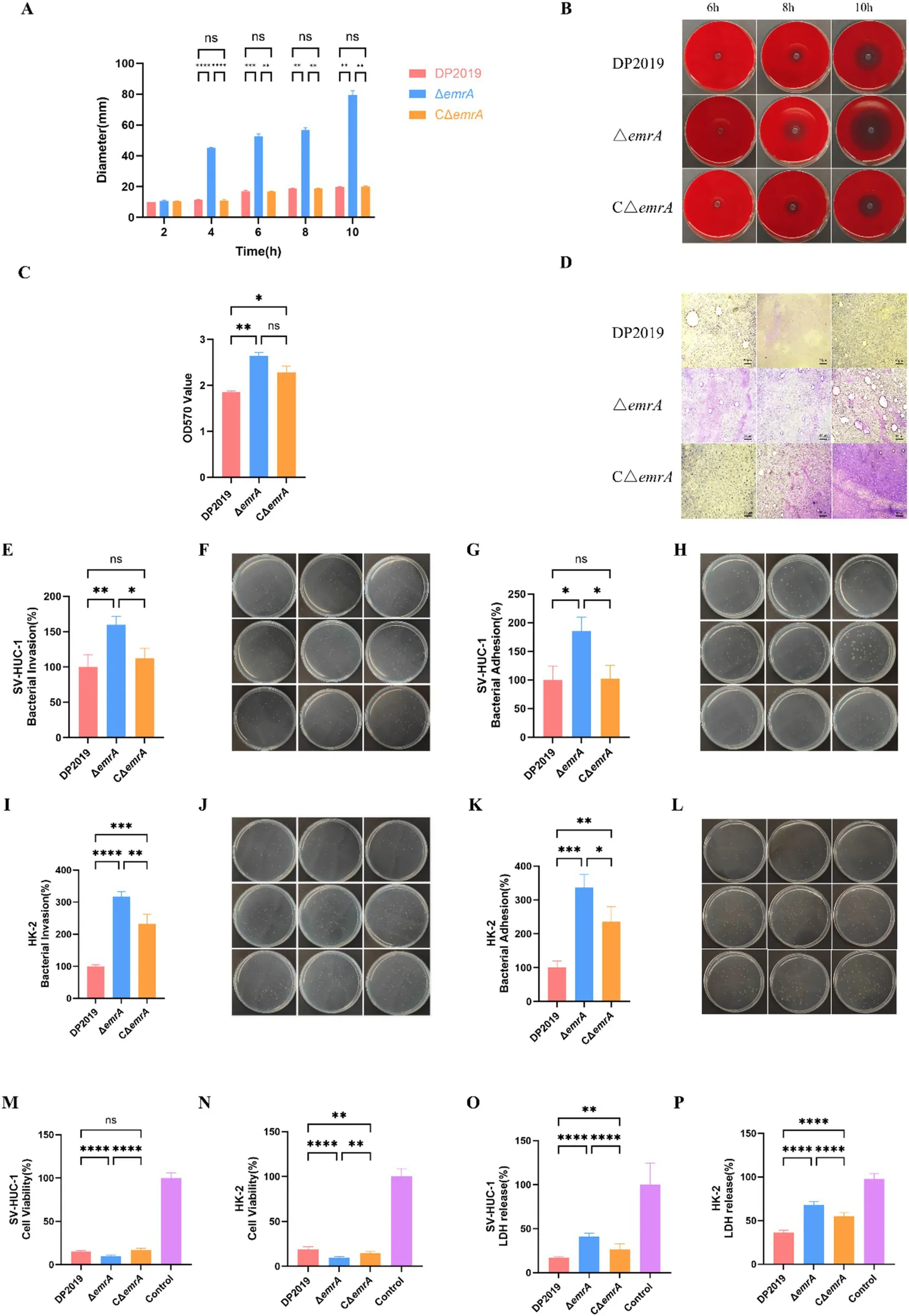
Effects of *emrA* deletion on swarming motility, biofilm formation, and host cell interactions. **(A,B)** Swarming motility of *P. mirabilis* WT (DP2019), Δ*emrA* mutant, and CΔ*emrA* on blood agar plates. **(A)** Quantification of swarming diameters (cm) **(B)** Representative images of swarming rings after 24 h of incubation. Data are presented as mean ± SD (*n* = 3 independent experiments). **(C,D)** Biofilm formation capacity after 24 h of static culture. **(C)** Quantification of biofilm biomass measured as optical density at 595 nm (OD570). **(D)** The three panels of each strain show representative wells after crystal violet staining, respectively. Data are shown as mean ± SD (*n* = 3 independent experiments). **(E–L)** Adhesion and invasion assays using SV-HUC-1 human urothelial cells and HK-2 human kidney proximal tubular epithelial cells. Bacterial adhesion **(E,G)** and invasion **(I,K)** were determined by plate counting (log_10_ CFU/well). The relative adhesion rates **(F,H)** and relative invasion rates **(J,L)** were calculated by normalizing the values of the WT (DP2019) group to 100%. Data are presented as mean ± SD (*n* = 3 independent experiments). **(M,N)** Cell viability assessed by CCK-8 assay after infection of SV-HUC-1 **(M)** and HK-2 **(N)** cells with the indicated strains. Viability of uninfected control cells was set to 100%. Data are shown as mean ± SD (*n* = 3). **(O,P)** Lactate dehydrogenase (LDH) release in the supernatants of SV-HUC-1 **(O)** and HK-2 **(P)** cells after bacterial infection. The LDH level of the WT (DP2019) group was set to 100% to calculate relative release rates. Data are presented as mean ± SD (*n* = 6). Statistical analysis: All experiments were performed at least three independent times. Comparisons among three or more groups were performed using one-way ANOVA followed by Tukey’s post-hoc test. Significance levels are indicated as: * *P* < 0.05, ** *P* < 0.01, *** *P* < 0.001; n.s., not significant.

### EmrA deletion is associated with enhanced cellular pathogenicity *in vitro*

The virulence-associated phenotype of the Δ*emrA* mutant was evaluated using human urothelial cell lines. Compared to the WT and CΔ*emrA*, the Δ*emrA* mutant exhibited significantly enhanced adhesion to and invasion of both SV-HUC-1 (bladder) and HK-2 (kidney) cells (Figures 2E–L). Cytotoxicity experiments (CCK-8 viability and LDH release assays) further indicated that Δ*emrA* infection led to reduced host cell survival and increased cell membrane damage (Figures 2M–P). These data suggest that *emrA* deletion enhances several *in vitro* cell-associated pathogenic phenotypes of *P. mirabilis*.

### The Δ*emrA* mutant shows enhanced virulence-associated phenotypes and exacerbated tissue damage *in vivo*

A rat model of ascending urinary tract infection was employed to assess the *in vivo* phenotype associated with *emrA* deletion. Results showed that the bacterial loads in the bladder and kidneys of rats in the Δ*emrA* infection group were significantly higher than those in the WT (DP2019) and CΔ*emrA* group (Figures 3A,B). Accompanied by significantly elevated levels of the pro-inflammatory cytokines (IL-1β, IL-6, and TNF-α) in the bladder tissues of Δ*emrA*-infected rats (Figures 3C–E). Histopathological analysis revealed that Δ*emrA* infection caused bladder epithelial exfoliation, edema, and extensive inflammatory cell infiltration, whereas the WT and CΔ*emrA* groups showed only mild focal inflammation (Figure 3H). Immunohistochemistry confirmed strong, widespread expression of IL-6 and TNF-α in the bladders of Δ*emrA*-infected group (Figures 3F,G,I,J). The infection with the Δ*emrA* mutant was also associated with significant renal dysfunction, manifested as elevated serum creatinine and urea levels, indicating impaired glomerular filtration (Figures 3K,L). Concurrently, serum magnesium and phosphorus levels were significantly decreased (Figures 3M,N). Histology of kidney tissues from the mutant group showed epithelial shedding and interstitial inflammation, consistent with the biochemical evidence of injury (Figure 3O).

**Figure 3:**
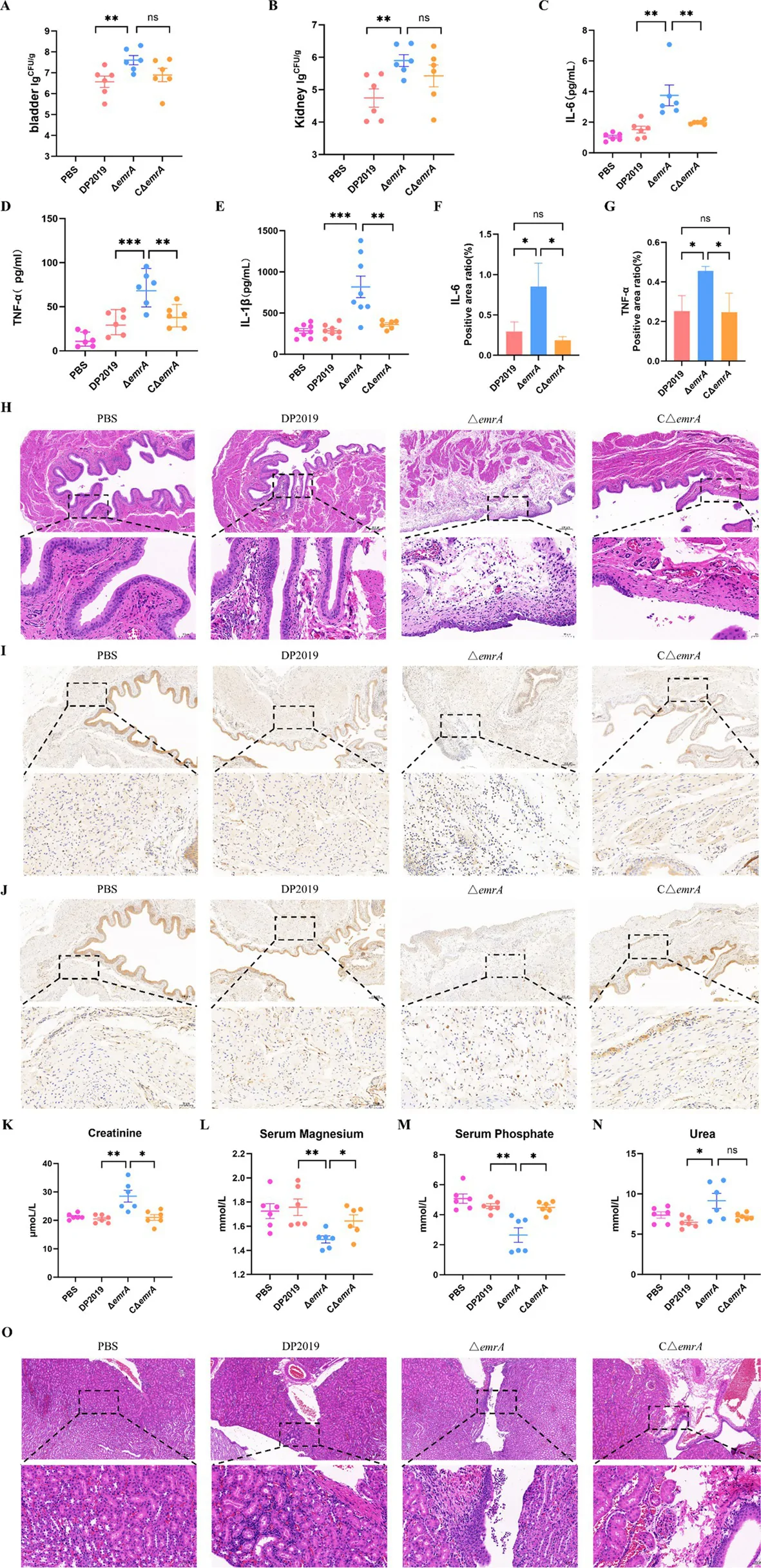
*In vivo* pathogenicity of the Δ*emrA* mutant in a rat model of urinary tract infection. **(A,B)** Colonization capacity of *P. mirabilis* strains in rat bladder **(A)** and kidney **(B)** tissues. Bacterial loads are expressed as log_10_ CFU per gram of tissue. Each symbol represents an individual animal (*n* = 6 per group). Horizontal lines indicate the median. **(C–E)** Levels of pro-inflammatory cytokines in bladder tissue homogenates measured by ELISA: IL-6 **(C)**, IL-1β **(D)**, and TNF-α **(E)**. Data are presented as pg./mg total protein, mean ± SD (*n* = 6 per group). **(H)** Representative hematoxylin and eosin (H&E) staining of bladder tissue sections from rats infected with the indicated strains. Scale bar = 200 μm (left panels) and 50 μm (right panels). **(F,G,I,J)** Immunohistochemical analysis of IL-6 and TNF-α expression in bladder tissue sections. **(F,G)** Quantitative analysis of IL-6-positive area (%) and TNF-α-positive area (%). **(I,J)** Representative images of IL-6 and TNF-α staining (brown signal). Data are presented as mean ± SD (*n* = 5 random fields per section from 3 rats per group). **(K–N)** Serum levels of biochemical markers indicative of renal function and electrolyte balance. **(K)** Creatinine (μmol/L). **(L)** Magnesium (mmol/L). **(M)** Phosphorus (mmol/L). **(N)** Urea (mmol/L). Each symbol represents an individual animal (*n* = 6 per group). Horizontal lines indicate the median. **(O)** Representative hematoxylin and eosin (H&E) staining of kidney tissue sections from rats infected with the indicated strains. Scale bar = 200 μm (left panels) and 50 μm (right panels). Statistical analysis: Normality of the data was assessed using the Shapiro–Wilk test. For non-normally distributed data (bacterial loads, cytokine levels, and IHC quantification), pairwise comparisons between groups were performed using the Mann–Whitney U test. For normally distributed data, one-way ANOVA followed by Tukey’s post-hoc test was applied. All tests were two-tailed. Significance levels are indicated as: * *P* < 0.05, ** *P* < 0.01, *** *P* < 0.01; n.s., not significant. For IHC data, * indicates a statistically significant difference compared to the DP2019 (WT) group (*P* < 0.05).

Notably, macroscopic bladder stones were observed exclusively in rats infected with the Δ*emrA* mutant. The urine from these animals was significantly more alkaline, and the characteristic decrease in serum magnesium and phosphorus—a biochemical hallmark of active struvite stone formation—provided further corroboration. Collectively, these *in vivo* data demonstrate that EmrA deletion is associated with increased urinary tract colonization, tissue damage and a microenvironment conducive to infection stone formation.

### The enhanced ability of *Proteus mirabilis* to form stones after EmrA deficiency is related to the increased urease activity

To evaluate the association between EmrA and infection stone-related phenotype, we performed complementary *in vitro* analyses. *In vitro* crystallization experiments showed that the crystal nucleation and aggregation abilities of the Δ*emrA* mutant were significantly enhanced compared with the WT and CΔ*emrA* (Figures 4A–D). This pro-calculogenic phenotype was linked to a fundamental increase in ureolytic activity. Further detection revealed that Urease activity of the Δ*emrA* mutant, measured by multiple independent assays (measured via pH changes, phenol red colorimetry, indophenol blue method), was significantly higher than that of WT and CΔ*emrA* (Figures 4E–G). At the molecular level, RT-qPCR analysis revealed that the mutant exhibited upregulated expression of key urease-associated genes, including the structural subunits (*ureA*, *ureB*, *ureC*), accessory proteins (*ureE*, *ureG*), and the transcriptional activator *ureR* (Figures 4H,I).

**Figure 4:**
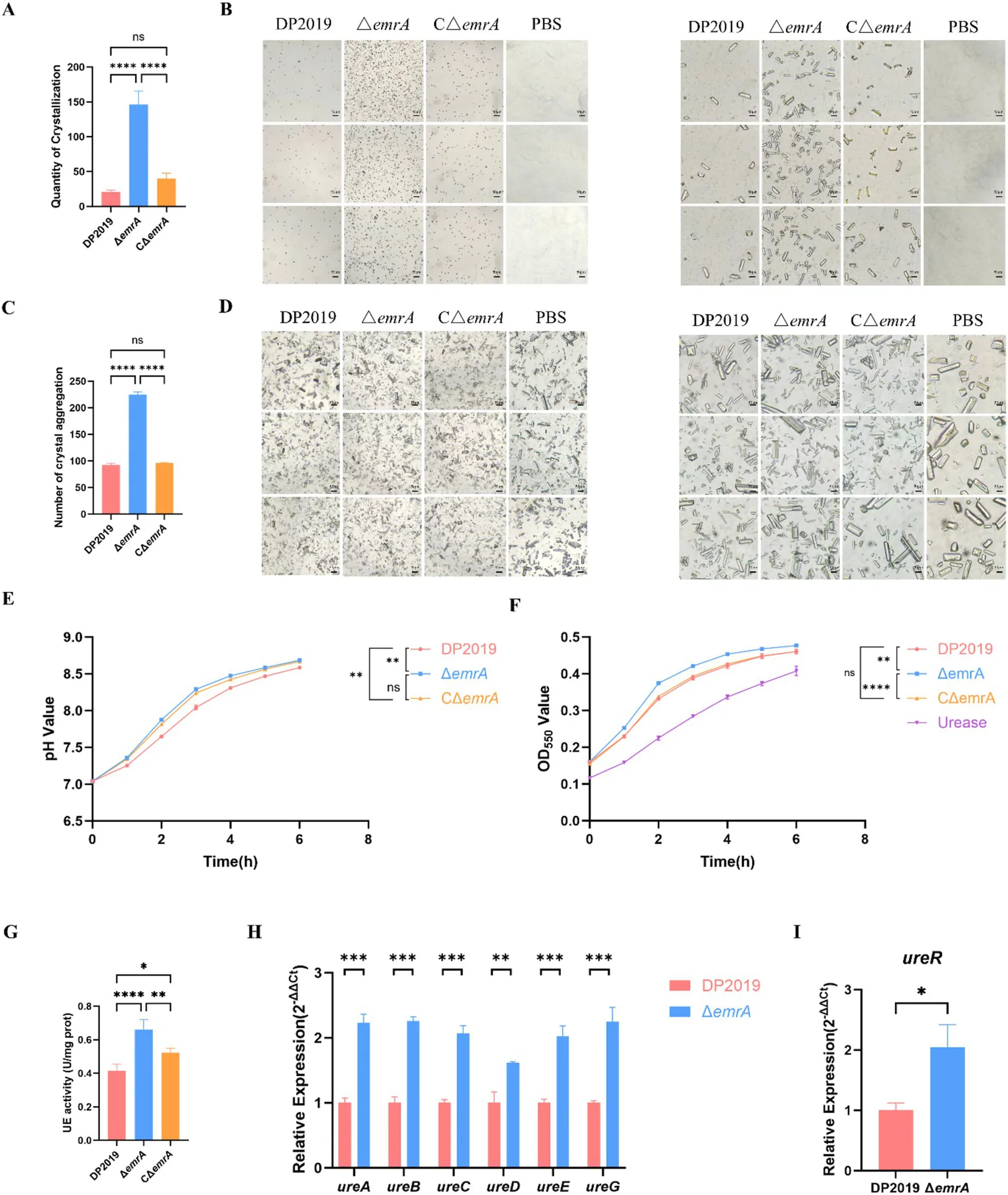
EmrA deficiency enhances crystal formation, aggregation and urease activity in *P. mirabilis*. **(A,B)** Crystal formation assay. **(A)** Quantification of crystal formation in artificial urine. **(B)** Representative microscopic images of crystals formed by each strain (scale bar = 50 μm). **(C,D)** Crystal aggregation assay. **(C)** Quantification of crystal aggregation. **(D)** Representative images of crystal aggregates. **(E–G)** Urease activity measured by three independent methods. **(E)** pH increase over time in urea-containing medium. **(F)** Phenol red colorimetric assay (OD550). **(G)** Indophenol blue colorimetric method, quantifying ammonia production. All data are presented as mean ± SD (*n* = 3 independent experiments). **(H,I)** Relative expression levels of urease subunit genes and the transcriptional regulator *ureR* determined by quantitative real-time PCR (qPCR). Expression levels were normalized to the housekeeping gene (*gurB*) and are shown relative to the WT (DP2019) strain, which was set to 1. Data are presented as mean ± SD (*n* = 3 biological replicates). Statistical analysis: Normality of the data was assessed using the Shapiro–Wilk test. Comparisons among three or more groups were performed using one-way ANOVA followed by Tukey’s post-hoc test. Between-group differences were assessed using an independent-samples *t*-test. Significance levels are indicated as: * *P* < 0.05, ** *P* < 0.01, *** *P* < 0.001; n.s., not significant.

### EmrA deficiency is associated with increased stone formation in a chronic infection model

A rat zinc disc implantation model was used to assess stone formation during chronic infection. The results showed that the stone mass of the Δ*emrA* mutant infection group showed an increasing trend compared to the WT group, and was significantly higher than that of the CΔ*emrA* group (Figures 5A,B). This enhanced stone burden was associated with significantly higher bacterial loads in the bladder, kidneys, and urine of rats infected with the Δ*emrA* mutant, consistent with enhanced colonization and persistence (Figures 5C–E). Analysis of the host response in this chronic model revealed a distinct profile. Contrary to the acute model, chronic ΔemrA infection showed reduced bladder and serum IL-1β, IL-6, and TNF-α despite high bacterial loads (Figures 5F–I). This discrepancy may reflect the inflammatory peak may have already subsided in the chronic phase or prolonged bacterial persistence might drive immune exhaustion or modulation. Meanwhile, the formation of stones may physically isolate bacteria from immune cells, which can also affect the occurrence of immune responses.

**Figure 5:**
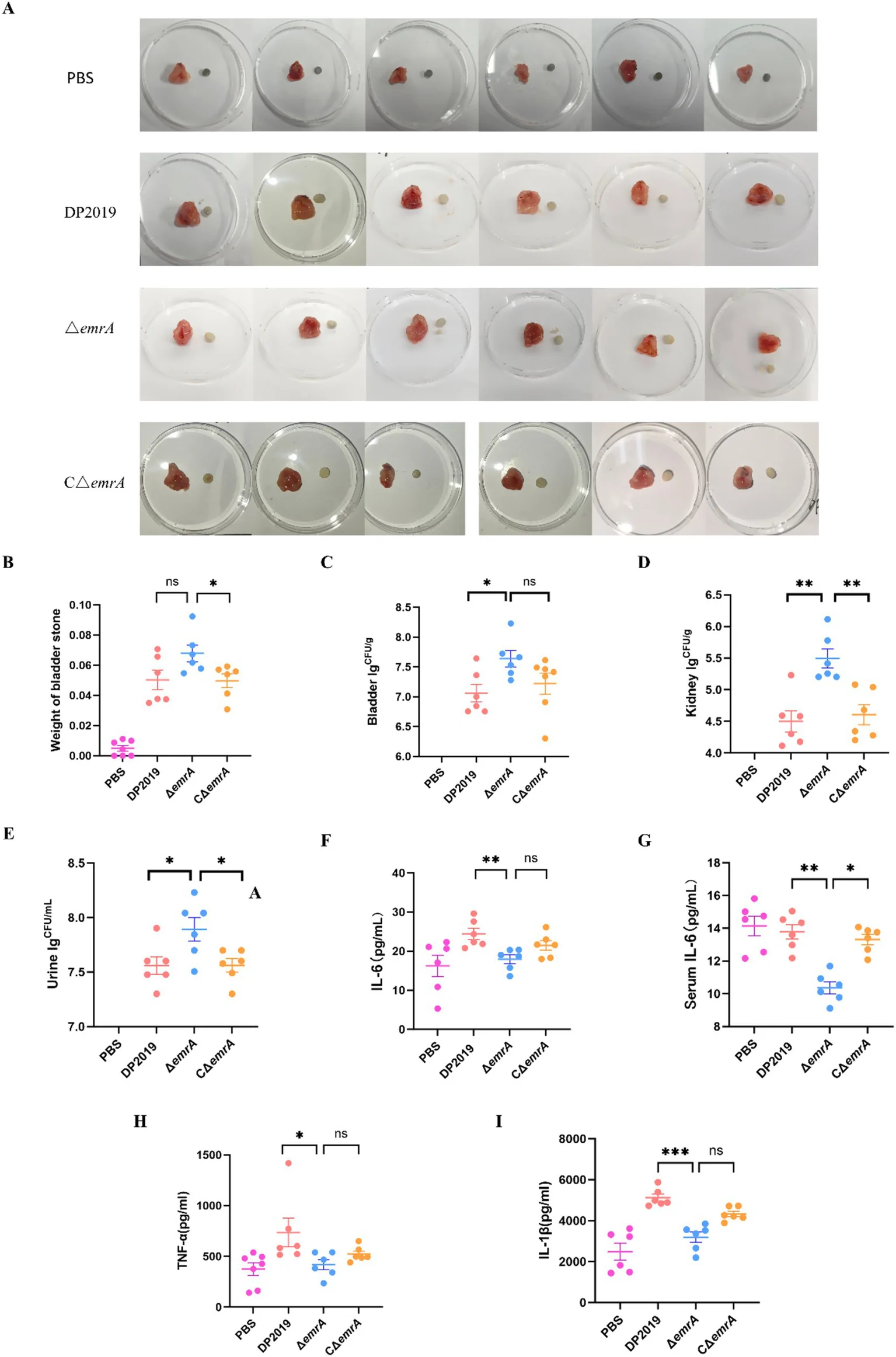
EmrA deficiency promotes bladder stone formation, enhances bacterial colonization, and modulates inflammatory responses in a chronic infection stone model. **(A,B)** Bladder stone formation in rats infected with *P. mirabilis* strains. **(A)** Representative images of bladder stones retrieved from rats infected with WT (DP2019), Δ*emrA* mutant, or complemented (CΔ*emrA*) strain. **(B)** Quantification of stone weight (mg). Each symbol represents an individual animal (*n* = 6 per group). Horizontal lines indicate the median. **(C–E)** Bacterial colonization capacity. Bacterial loads in bladder tissue **(C)**, kidney tissue **(D)** and urine **(E)** are expressed as log₁₀ CFU per gram of tissue or per mL of urine. Each symbol represents an individual animal; horizontal lines indicate the median. **(F–I)** Inflammatory cytokine levels. **(F)** IL-6, **(G)** IL-1β, and **(H)** TNF-α in bladder tissue homogenates (pg/mg total protein). **(I)** Serum IL-6 levels (pg/mL). Each symbol represents an individual animal; horizontal lines indicate the median. Statistical analysis: Normality of the data was assessed using the Shapiro–Wilk test. As the data were not normally distributed, pairwise comparisons between groups (Δ*emrA* vs. WT, Δ*emrA* vs. CΔ*emrA*, and WT vs. CΔ*emrA*) were performed using the Mann–Whitney *U* test. All tests were two-tailed. Significance levels are indicated as: * *P* < 0.05, ** *P* < 0.01, *** *P* < 0.001; n.s., not significant.

### Transcriptomic profiling identifies expression changes associated with EmrA deletion

To explore pathways associated with the Δ*emrA* mutant phenotypes, we conducted comparative transcriptomic analysis (RNA-seq). Sample-level expression distributions were first evaluated (Figure 6A). Basic functional annotation analysis was performed based on the reference genome GCF_026167565.1. RNA-seq analysis showed that *emrA* deletion led to differential expression of 134 genes (|log₂FC| ≥ 1, padj <0.05), including 54 upregulated and 80 downregulated genes (Figure 6B). Functional enrichment analysis revealed that upregulated genes were primarily enriched in virulence-related pathways. Key virulence-associated operons were significantly induced, including genes encoding the Type VI secretion system (*tssB*, t*ssC*, *tssF*), fimbrial components (*fimA*), and hemolysin (*hpmB*). Correspondingly, Gene Ontology (GO) and KEGG pathway analyses highlighted significant enrichment for biological processes related to fimbrial assembly, cell adhesion, biofilm formation, and protein secretion (Figures 6C–F). The urease subunit genes (*ureA*, *ureB*, *ureC*, *ureE*, *ureG*) showed an upregulation trend but did not reach a statistically significant level (padj>0.05). In contrast, downregulated genes were predominantly enriched in core metabolic pathways, most notably in amino acid biosynthesis and metabolism. Genes involved in arginine synthesis (*argA*, *argB*, *argC*, *argF*) and transport (*artP*) were significantly suppressed (Figures 6G–J). This transcriptional signature indicates that EmrA deletion is associated with changes in bacterial resource allocation, and is associated with virulence gene expression while inhibiting basic metabolism.

**Figure 6:**
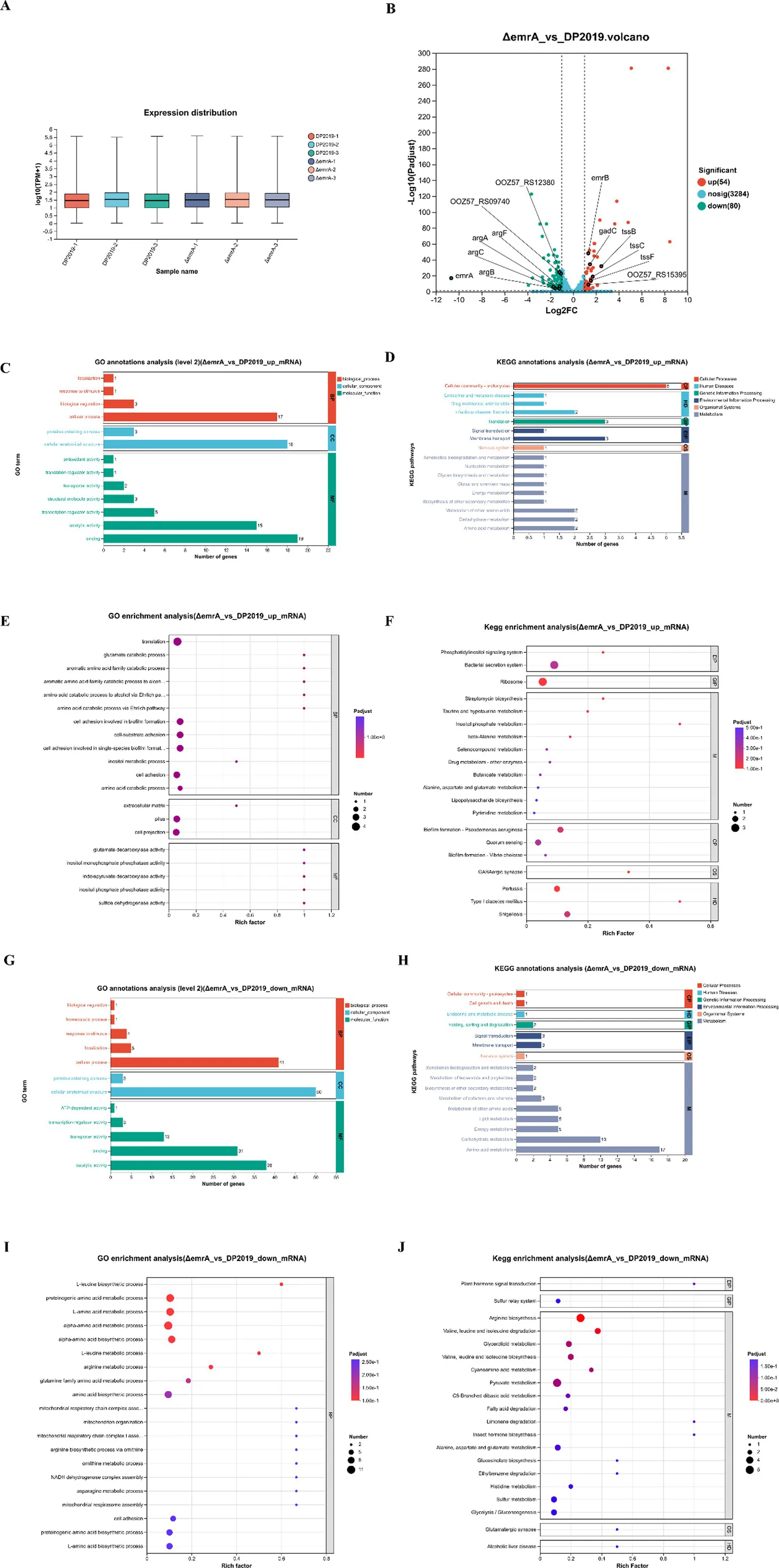
Transcriptomic profiling of the mutant compared to the WT strain. **(A)** Box plot showing the distribution of gene expression levels (*log₁₀*(TPM + 1)) in WT and Δ*emrA* samples. The boxes represent the interquartile range (IQR), with the line inside each box indicating the median. Whiskers denote the 5th and 95th percentiles. **(B)** Volcano plot of differentially expressed genes (DEGs). The *x*-axis represents *log2*(fold change) (*log2FC*) between Δ*emrA* and WT, and the *y*-axis represents −*log10*(adjusted *p*-value). Red dots: Significantly upregulated genes (*log2FC* ≥ 1, adjusted *p* < 0.05). Green dots: Significantly downregulated genes (*log2FC* ≤ −1, adjusted *p* < 0.05). Blue dots: Non-significant genes. **(C,D)** Functional annotation of upregulated DEGs. **(C)** GO annotation analysis (level 2) showing the distribution of upregulated DEGs across biological process, cellular component, and molecular function categories. **(D)** KEGG annotation analysis showing the top enriched pathways for upregulated DEGs. **(E,F)** Enrichment analysis of upregulated DEGs. **(E)** GO enrichment analysis displaying significantly enriched GO terms. **(F)** KEGG enrichment analysis displaying significantly enriched KEGG pathways. **(G,H)** Functional annotation of downregulated DEGs. **(G)** GO annotation analysis (level 2) showing the distribution of downregulated DEGs across biological process, cellular component, and molecular function categories. **(H)** KEGG annotation analysis showing the top enriched pathways for downregulated DEGs. **(I,J)** Enrichment analysis of downregulated DEGs. **(I)** GO enrichment analysis displaying significantly enriched GO terms. **(J)** KEGG enrichment analysis displaying significantly enriched KEGG pathways. Statistical analysis: Differential expression analysis was performed using DESeq2 (V1.42.0). Genes with |*log2*(fold change)| ≥ 1 and an adjusted *p*-value (Benjamini-Hochberg correction) < 0.05 were considered significantly differentially expressed. For GO and KEGG enrichment analyses, significance was defined as an adjusted *p*-value < 0.05 (Benjamini-Hochberg corrected). All RNA-seq experiments were performed with three biological replicates per condition.

## Discussion

In this study, we investigated the association of the membrane fusion protein EmrA of *P. mirabilis* with antimicrobial susceptibility, stress adaptation, multiple virulence-associated phenotypes, and infection stone formation in *P. mirabilis*. Using an isogenic *emrA* deletion mutant, complemented strain, *in vitro* phenotypic assays, transcriptomic profiling, and rat infection models, we found that deletion of *emrA* decreased MICs for carbenicillin and ampicillin, increased the MIC for tetracycline, and left most tested antibiotics unchanged; *emrA* deletion also displayed impaired adaptation to nutrient limitation, heat, and acidic stress conditions, but increased survival under oxidative-stress; In addition, *emrA* deletion was associated with enhanced swarming motility, biofilm formation, host-cell adhesion and invasion, and urease activity, and infection stone-associated phenotypes. In a rat model of urinary tract infection and an infectious stone model, the mutant was associated with more severe bladder and kidney colonization, a stronger inflammatory response and increased bladder struvite stone formation. Transcriptome analysis revealed that EmrA deficiency upregulated virulence gene clusters such as T6SS, fimbriae, hemolysin, urease, stress-response, and metabolic genes. While downregulating universal stress protein genes. The complemented strain often shifted phenotypes toward WT, supporting involvement of *emrA*. Together, this complex profile suggests that EmrA may shape the balance among membrane transport, stress physiology and virulence-associated programs rather than controlling a single linear resistance pathway, although the underlying regulatory mechanisms remain to be defined.

Bacteria exposed to antibiotic and host immune pressures may display resource-based or regulatory trade-off between antimicrobial resistance and virulence (Beceiro et al., 2013). In the present study, comparison of the WT and Δ*emrA* strains showed that *emrA* deletion was associated with lower MICs for ampicillin and carbenicillin, a higher MIC for tetracycline, while most other antibiotics were unaffected. This pattern indicates an antibiotic-specific susceptibility phenotype rather than a generalized change in antimicrobial resistance. However, these findings should not be interpreted as definitive evidence that EmrA directly mediates resistance to ampicillin or carbenicillin, because restoration of the MIC phenotype was not evaluated in CΔ*emrA*. Beyond antimicrobial susceptibility, the Δ*emrA* mutant grew comparably to WT in nutrient-rich LB medium but displayed reduced growth under nutrient-limited and oxygen-limited conditions and reduced survival following heat and acid stress. In contrast, survival under oxidative-stress was increased. These condition-dependent responses suggest that *emrA* genotype is associated with altered stress adaptation rather than a generalized increase or decrease in bacterial fitness. Such environmental conditions may be relevant to the urinary tract infection, where bacteria can encounter nutrient restriction, variable oxygen tension, acidic microenvironments, and host-derived oxidative stress. The Δ*emrA* mutant also exhibited enhanced virulence-associated phenotypes. We therefore hypothesize that the physiological changes associated with *emrA* deletion may shift the balance between selected stress-adaptation functions and virulence-associated traits. This interpretation remains provisional because the underlying regulatory mechanism has not been established. Context-dependent relationships between antimicrobial resistance determinants and virulence have also been reported in other bacteria. For example, loss of the porins OmpC/OmpD in *Enterobacter hormaechei* confers carbapenem resistance but severely attenuates virulence (Perault et al., 2025). The *cfr*-like genes drive linezolid resistance while simultaneously impairing sporulation, biofilm formation, and toxin production in *C. difficile*, illustrating a resistance-virulence association. Similar links between efflux systems and virulence-associated physiology have been described in other pathogens, including *Pseudomonas aeruginosa*, deletion of the MexAB-OprM efflux pump also affects quorum sensing and virulence gene expression (Alcalde-Rico et al., 2020). These studies provide conceptual evidence that resistance-associated systems can influence broader bacterial physiology, but they do not establish the mechanism operating in *P. mirabilis*. The coexistence of reduced MICs for selected β-lactam antibiotics and enhanced virulence-associated phenotypes in the Δ*emrA* mutant is consistent with a possible resistance–virulence association. However, the current findings do not establish a genetically validated mechanistic link between the antibiotic-susceptibility phenotype and virulence-associated changes, because the antibiotic-susceptibility phenotype was evaluated only in the WT and deletion strains. The increased oxidative-stress tolerance of the mutant may partly explain its enhanced persistence *in vivo*, whereas its reduced heat and acid tolerance indicates that *emrA* deletion does not globally increase stress resistance. Additional genetic controls and mechanistic experiments will be required to determine whether and how EmrA links antimicrobial susceptibility, stress adaptation, and virulence-associated physiology.

Our study found that *emrA* deficiency is associated with enhanced virulence-associated phenotypes in *P. mirabilis*. Swarming motility is a key mechanism for the retrograde spread of *P. mirabilis* from the bladder to the kidneys, and its enhancement may facilitate bacterial colonization of the bladder from the urethra and subsequent ascent to the kidneys. Biofilms serve as a protective barrier for bacteria against host immunity and antibiotic attack; enhanced biofilm formation increases bacterial colonization efficiency on catheter surfaces and the urothelium, thereby promoting resistance to antibiotic killing and immune clearance and serving as a source of recurrent infection. Our results showed that the mutant exhibited significantly increased swarming migration on solid media and enhanced biofilm formation. This regulatory pattern has been validated in other bacteria. In *E. coli*, deletion of multiple TolC-dependent efflux pumps, including EmrAB-TolC, resulted in increased swimming motility except Δ*emrY* and Δ*acrZ* (Di Maso and Ruiz, 2024). Moreover, it was observed that in the presence of cationic antimicrobial compounds, deletion mutants of several secondary multidrug efflux pumps, including EmrA, displayed significantly increased biofilm formation (Bay et al., 2017). These studies indicate that efflux pump components potentially influence bacterial motility and biofilm formation.

Adhesion and invasion are critical steps in bacterial infection of host cells. Adhesion is a prerequisite for bacterial colonization of the urothelium, while invasion allows bacteria to evade host immune clearance and cause deep tissue damage. Our study showed that the mutant exhibited significantly higher adhesion and invasion rates to both SV-HUC-1 and HK-2 urothelial cells compared to the WT and CΔ*emrA*, suggesting that *emrA* deletion may enhance bacterial-host cell interactions through altered expression of fimbriae, adhesins or other surface-associated factors. Cytotoxicity assays using CCK-8 and LDH release confirmed that the survival of urothelial cells treated with the knockout strain was significantly decreased, while LDH release was increased, indicating that *emrA* deletion enhances the direct damage capability of bacteria to host cells. To validate the physiological relevance of these *in vitro* phenotypes, we established a rat model of urinary tract infection. The results showed that *emrA* deletion was associated with enhanced *in vivo* pathogenicity-associated phenotypes. The bacterial loads of the knockout strain in bladder and kidney tissues were significantly higher than those of the WT and CΔ*emrA*, indicating that EmrA loss is associated with increased bacterial colonization and proliferation in the urinary tract, thereby laying the foundation for subsequent infection progression. In the mutant group, the levels of pro-inflammatory cytokines such as IL-6, IL-1β and TNF-α in bladder tissue homogenates were significantly elevated. Histopathological examination (HE staining) revealed more severe inflammatory cell infiltration and epithelial damage and immunohistochemistry further confirmed the local enrichment of inflammatory factors, suggesting that *emrA* deletion is associated with exacerbates inflammatory damage to the urinary tract mucosa either by enhancing bacterial pathogenicity or by directly modulating the host immune response. Furthermore, serum creatinine and urea levels were significantly increased in the mutant group, indicating impaired renal function. Together, these results demonstrate that EmrA deficiency is associated with more severe pathological outcomes of urinary tract infection by enhancing *in vivo* colonization capacity and pro-inflammatory effects. EmrA is associated with the virulence in *P. mirabilis* and its deletion results in a consistent virulence gain effect across the entire chain from motility and colonization to tissue damage.

Combined with transcriptomic sequencing results, we observed that multiple virulence-related genes were significantly upregulated in the mutant strain, which may contribute to the enhanced virulence-associated phenotypes. First, the expression of core components of the Type VI secretion system (T6SS), including *tssB*, *tssC*, *tssF* and Vgr family protein genes, was upregulated in the mutant strain, consistent with a molecular explanation for the increased cytotoxicity. T6SS is a contact-dependent killing apparatus that can directly disrupt host cell membrane integrity through perforin-like effector proteins, and its activation leads to increased LDH release and reduced cell viability (Reglinski et al., 2025). Second, potential fimbriae genes were significantly upregulated, consistent with a structural basis for the enhanced adhesion and invasion. Fimbriae are the primary tools for bacterial adhesion to host cells, and their increased expression may improve binding efficiency to receptors on urothelial cells, thereby promoting invasion and initial biofilm formation (Gahlot et al., 2022). In addition, upregulation of the haemolysin accessory protein gene *hpmB* suggests enhanced haemolysin activity, which together with T6SS may further aggravate cell damage (Uphoff and Welch, 1990). The coordinated activation of these virulence genes partially explains at the molecular level the phenotypes of increased adhesion/invasion, elevated cytotoxicity and enhanced biofilm formation after EmrA deletion.

Infectious stones are a serious complication of *P. mirabilis* infection, and their formation depends on urease-mediated urea hydrolysis and urine alkalinisation (Fitzgerald et al., 2024). In this study, the mutant strain exhibited significantly enhanced capacity in both crystal nucleation and aggregation assays, along with markedly increased urease activity. Mechanistically, this was attributed to upregulation of the urease transcriptional activator UreR and the urease structural genes. Although the transcriptomic data revealed a similar trend for urease-encoding genes, the increase did not reach statistical significance, which we attribute to differences in detection sensitivity between the two methods. This finding is consistent with EmrA to the transcriptional regulation of urease, whereas previously the activation of the urease operon was known to rely mainly on UreR and nitrogen sensing. These results suggest a possible relationship between EmrA and the urease system, but they do not demonstrate direct transcriptional regulation. It should be noted that the crystal formation and aggregation assays were performed using bacterial lysates rather than live bacteria, which may not fully reflect the dynamic physiological processes, such as active metabolism and continuous urease production, that occur during *in vivo* infection and stone formation. But, in a chronic infectious stone model, the stones formed by the mutant showed a certain upward trend compared to WT and CΔ*emrA*, supporting an association with the *in vitro* findings without establishing a direct causal pathway. Of note, in this chronic model, the mutant group displayed a lower local inflammatory response than the WT group, despite higher bacterial loads. This seemingly paradoxical phenomenon may reflect immune exhaustion induced by persistent infection with a higher bacterial burden, or a physical barrier effect resulting from extensive stone formation (Weißelberg et al., 2024), suggesting that EmrA deficiency not only enhances stone-forming capacity but may also promote chronic infection by reshaping the host immune response. This apparent discrepancy between enhanced virulence-associated phenotypes and attenuated inflammation precisely illustrates the complexity of bacterial resource reallocation strategies and reminds us that different dimensions of pathogenicity should be distinguished when evaluating bacterial virulence.

Further analysis of the transcriptomic data revealed that, in contrast to the significantly upregulated virulence genes, several metabolism- and stress-related genes were markedly downregulated in the mutant strain. Downregulation of the arginine synthesis pathway and the arginine transporter gene *artP* suggests that the mutant strain may be associated with reduced resource investmentin energy-consuming amino acid synthesis. Moreover, significant downregulation of the universal stress protein genes *OOZ57_RS09740*(KO name: *uspG*) and *OOZ57_RS12380* (KO name: *uspF*) reveals a dual role for EmrA in environmental adaptation. Universal stress proteins are induced under various environmental stresses such as acid, heat and nutrient starvation (Zhang et al., 2025; Weißelberg et al., 2024). Therefore, their downregulation may explain the reduced survival of the knockout strain under heat and acid stress. The paradoxical phenotype of increased oxidative stress tolerance suggests that compensatory activation of the antioxidant system may occur independently of the regulation of universal stress proteins, a mechanism that warrants further investigation. Arginine is a precursor for polyamine synthesis, and polyamines are involved in biofilm formation and motility regulation (Liu et al., 2022; Scribani Rossi et al., 2022; Shi et al., 2019). However, the mutant strain did not show reduced biofilm or motility. On the contrary, these were significantly enhanced. We propose the following explanations. First, the marked upregulation of T6SS core components, fimbriae genes and the haemolysin accessory protein may directly enhance bacterial adhesion, aggregation and surface migration, and this positive driving effect overcomes any potential negative impact of reduced polyamines. Second, bacteria may maintain basal polyamine levels through compensatory activation of the ornithine decarboxylase pathway or exogenous polyamine uptake to support virulence functions. Finally, the downregulation of the arginine pathway reflects a resource redistribution strategy that actively reduces energy intensive arginine synthesis and reinvests resources in the effective production of virulence factors, which may reflect a resource redistribution strategy associated with enhanced virulence gene expression (Kratz and Banerjee, 2024; Whittle et al., 2024). This seemingly contradictory phenomenon precisely illustrates the complexity of global transcriptional reprogramming in the knockout strain, which is associated with enhanced virulence-associated phenotypes through differential gene expression. This resource reallocation strategy is consistent with the possible association between antibiotic susceptibility and virulence-associated phenotypes. In summary, the transcriptomic data identify gene-expression changes associated with *emrA* deletion and provide a basis for mechanistic hypotheses. However, these data do not establish direct regulation or a complete molecular pathway, which will require targeted biochemical investigation and further genetic validation.

Traditional antibiotics impose strong selective pressure and can promote resistance spread (Ayres, 2024; Lu et al., 2025). Anti-virulence strategies have been proposed as an approach that aims to attenuate pathogens without directly challenging their survival, potentially reducing the selection for resistance (Hopkins et al., 2024). Our observation that *emrA* deletion enhances virulence-associated phenotypes raises the question of whether maintaining EmrA function could, in principle, be explored as a concept for limiting pathogenicity. However, this idea remains entirely speculative and would require substantial experimental validation—including gain-of-function studies and direct mechanistic investigation—before any therapeutic implications could be considered. Therefore, we do not propose EmrA as a target for therapeutic development at this stage, but rather highlight the association as a starting point for future mechanistic research.

The direct interaction between EmrA and known virulence-associated regulatory proteins has not been clarified. While transcriptomic data revealed differential expression of multiple virulence gene clusters, we have not yet identified the protein interaction partners of EmrA using methods such as co-immunoprecipitation, bacterial two-hybrid screening or crosslinking mass spectrometry. Whether EmrA directly binds UreR, TssB or other regulatory proteins, or acts through indirect pathways, remains unclear. Construction of deletion mutants of key regulators and examination of their genetic epistatic relationships with *emrA* will help to define the regulatory hierarchy in which EmrA operates. Furthermore, validation of the anti-virulence target is still at a conceptual stage. We have not performed small-molecule screening, nor have we constructed strains with conditional overexpression of *emrA* to assess the therapeutic effect of enhancing EmrA function. In addition, epidemiological surveys have not yet been conducted to determine whether natural mutations or polymorphisms in *emrA* exist in clinical isolates, and whether such mutations are associated with enhanced virulence or stone-forming capacity. In summary, this study reveals an association between EmrA and multiple phenotypic traits in *P. mirabilis*, including specific antibiotic susceptibility changes, stress adaptation, and virulence- and stone-associated phenotypes. EmrA deficiency is associated with reduced expression of stress-response genes and increased expression of virulence-associated genes, which may contribute to, leading to more severe infection and stone complications in animal models. Transcriptomic analysis identified gene expression changes associated with *emrA* deletion, involving pathways such as T6SS, fimbriae, and haemolysin, but the underlying molecular mechanisms remain to be determined. The observation that *emrA* deletion correlates with both reduced β-lactam susceptibility and enhanced virulence-associated phenotypes raises the question of whether the EmrAB-TolC system might be explored as a target for anti-virulence research. However, such a possibility remains hypothetical and would require substantial experimental validation, including gain-of-function studies and small-molecule screening, before any therapeutic implications can be considered. This study also acknowledges the current limitations of the evidence, particularly the lack of direct molecular interactions between EmrA and known virulence regulators, thereby pointing to specific directions for future mechanistic investigation. Overall, our findings expand the understanding of the biological functions of bacterial efflux pumps and provide a basis for further research into the role of EmrA in the pathogenesis of urinary tract infections.

## Conclusion

This study shows that *emrA* deletion is associated with antibiotic-specific susceptibility changes, altered stress responses, and enhanced virulence- and stone-associated phenotypes in *P. mirabilis*. Because the MIC phenotype was not tested in the complemented strain, the direct contribution of EmrA to ampicillin and carbenicillin resistance remains unresolved. These findings support further investigation of the *emrAB* locus but do not yet establish EmrA as a genetically validated regulator of antimicrobial resistance. Although EmrA appears to play a role in intrinsic resistance to some β-lactam antibiotics and in stress adaptation, its genetic inactivation was associated with a phenotype exhibiting enhanced virulence-associated traits, including increased swarming, biofilm formation, adhesion, invasion, urease activity, and stone formation. The combined evidence from phenotypic, transcriptomic, and animal studies suggests that the absence of EmrA is associated with a phenotype showing enhanced virulence-associated traits, with higher expression of virulence determinants and lower expression of stress-response genes. These observations provide additional insight into the pleiotropic effects associated with *emrA* deletion in *P. mirabilis*. The association between EmrA and virulence-associated phenotypes may warrant further investigation of the EmrAB-TolC system in the context of *P. mirabilis* pathogenesis, although any therapeutic implications remain entirely speculative at this stage.

## Data Availability

The datasets presented in this study can be found in online repositories. The names of the repository/repositories and accession number(s) can be found at: https://www.ncbi.nlm.nih.gov/, PRJNA1472729.
