# Supplementary material for: The impact of EmrA deficiency on antibiotic-susceptibility and virulence- and stone-associated phenotypes in Proteus mirabilis
Source: Front Microbiol. 2026 Sep 14;17:1895236. doi: 10.3389/fmicb.2026.1895236 (PMC13617128; doi:10.3389/fmicb.2026.1895236)
Supplement: Supplementary file 1 [file Supplementary_file_1.DOCX]

Figure S1. Transcriptional expression of *emrA* gene in the CΔ*emrA*

Using the expression level of *emrA* gene in WT as the control, analyze the relative expression level of genes in the CΔ*emrA* (n=3). The mean ± standard deviation of the WT and CΔ*emrA* are 1.005 ± 0.1189 and 0.5924 ± 0.1947, respectively.

Statistical analysis: Normality of the data was assessed using the Shapiro–Wilk test. Between‑group differences were assessed using an independent‑samples *t*‑test. Significance levels are indicated as: *$P$ < 0.05, **$P$ < 0.01, ***$P$ < 0.001; n.s., not significant.
